# Optical Tweezers as a New Biomedical Tool to Measure Zeta Potential of Stored Red Blood Cells

**DOI:** 10.1371/journal.pone.0031778

**Published:** 2012-02-21

**Authors:** Diego C. N. Silva, Cauêh N. Jovino, Carlos A. L. Silva, Heloise P. Fernandes, Milton M. Filho, Sheyla C. Lucena, Ana Maria D. N. Costa, Carlos L. Cesar, Maria L. Barjas-Castro, Beate S. Santos, Adriana Fontes

**Affiliations:** 1 Departamento de Biofísica e Radiobiologia, Universidade Federal de Pernambuco, Recife, Pernambuco, Brazil; 2 Departamento de Ciências Farmacêuticas, Universidade Federal de Pernambuco, Recife, Pernambuco, Brazil; 3 Hematology and Transfusion Center, Universidade Estadual de Campinas, INCTS-Instituto Nacional de Ciência e Tecnologia do Sangue, Campinas, São Paulo, Brazil; 4 Fundação Hemope, Recife, Pernambuco, Brazil; 5 Instituto de Física Gleb Wataghin, Universidade Estadual de Campinas, Campinas, São Paulo, Brazil; University of South Florida College of Medicine, United States of America

## Abstract

During storage, red blood cells (RBCs) for transfusion purposes suffer progressive deterioration. Sialylated glycoproteins of the RBC membrane are responsible for a negatively charged surface which creates a repulsive electrical zeta potential. These charges help prevent the interaction between RBCs and other cells, and especially among each RBCs. Reports in the literature have stated that RBCs sialylated glycoproteins can be sensitive to enzymes released by leukocyte degranulation. Thus, the aim of this study was, by using an optical tweezers as a biomedical tool, to measure the zeta potential in standard RBCs units and in leukocyte reduced RBC units (collected in CPD-SAGM) during storage. Optical tweezers is a sensitive tool that uses light for measuring cell biophysical properties which are important for clinical and research purposes. This is the first study to analyze RBCs membrane charges during storage. In addition, we herein also measured the elasticity of RBCs also collected in CPD-SAGM. In conclusion, the zeta potential decreased 42% and cells were 134% less deformable at the end of storage. The zeta potential from leukodepleted units had a similar profile when compared to units stored without leukoreduction, indicating that leukocyte lyses were not responsible for the zeta potential decay. Flow cytometry measurements of reactive oxygen species suggested that this decay is due to membrane oxidative damages. These results show that measurements of zeta potentials provide new insights about RBCs storage lesion for transfusion purposes.

## Introduction

RBCs have sialylated glycoproteins which are responsible for a negatively charged membrane cell surface [1]. In an electrolyte medium, such as in blood plasma, this induces the formation of a layer of surrounding medium ions of opposite charges rigidly bound around the cells, that creates a repulsive electrical zeta potential (ζ) between the RBCs. The ζ potential is an important property responsible to stabilize the RBCs colloidal suspension preventing cells to come too close and avoiding interactions between RBCs and other cells, and especially among themselves. In this way, the zeta potential not only regulates the adhesions among RBCs, but also between RBCs and endothelial cells, like cells from capillary walls [2]–[4]. Some authors have even reported a loss of sialic acid in mature cells and describe this loss as capable to decrease RBCs survival in circulation in animal models [5].

RBCs storage lesions can reduce post-transfusion RBCs survival in blood circulation [6]. To determine the reasons responsible for this, a number of investigators have examined changes in several biophysical and biochemical properties during storage. It is known that one of the most common already observed storage injuries is the loss of cell elasticity or deformability [7]. However, questions not answered yet are: RBCs membrane charges, consequently the ζ potential, can also change with storage time as well as the elasticity changes? If there are changes in ζ potential, what could be the cause? Elasticity and ζ potential can be somehow connected by some common factor? RBCs glycoproteins are sensitive to enzymes and can be even removed by them [8]. During RBCs storage period, the leukocyte degranulation promotes enzyme release, which can reduce glycoprotein membrane expressions and probably can change the membrane electrical charges [9]. This raises the question if the leukocyte enzymes are capable to change the ζ potential. The aim of this study was to search for answers on these issues by measuring the ζ potential of standard RBCs units and also of pre-storage leukocyte reduced RBCs units by using an optical tweezers (both samples were collected in CPD-SAGM and analyzed as a function of the storage time). We also measured RBCs elasticity, with an optical tweezers, and analyzed, by flow cytometry, the production of reactive oxygen species in standard stored RBCs units collected in the same preservative solution to correlate deformability with the ζ potential results.

Optical tweezers, a highly sensitive tool based on photon momentum transfer that uses an infrared laser beam tightly focused by the microscope objective [10], belong to the modern laser techniques that have shown a great contribution to optical microscopy and life sciences. Optical tweezers allow individual trapping and manipulation of biological systems, and can be used to obtain important properties of cells and molecules [11]–[16]. In particular, optical tweezers allow mechanical measurements of RBCs properties (such as the membrane viscosity and elasticity) of normal cells and also of cells altered by some external factors (for example: storage, ionizing radiation, action of a drug or even by hematological diseases) [17]–[20]. In addition, optical tweezers have recently been used to evaluate electrical properties of the membrane through measurements of zeta potential [21]. For single-cell manipulation, another modern and powerful tool, also based on light, which can be applied to evaluate cell deformability, is the optical stretcher. Recently, J. Guck and collaborators measured the elasticity of normal and malaria infected RBCs with an optical stretcher and showed that the evaluation of cell elasticity can be used to detect early stages of malaria infection with high sensitivity and speed [22]–[23].

In this paper, we show that ζ potential is an important property, as sensitive as elasticity to storage timing and conditions, which can provide new information about storage lesions for transfusion purposes. To our knowledge, this is the first study that monitors RBC membrane electrical charge changes with storage and the first time that the elasticity of RBCs collected in CPD-SAGM is analyzed and correlated to ζ potential. We quantified important RBCs parameters that can help in the comprehension of the effects caused in these cells by different blood storage conditions. We believe that the analysis of the changes of the electrical properties of RBCs membranes can also provide a better comprehension about cell senescence process and as well about hemagglutination reactions.

## Materials and Methods

### Optical Tweezers

The optical tweezers system consists of a laser beam in the near infrared (ζ = 1064 nm – IPG Photonics, EUA) focused on the microscope (Axiolab, Carl Zeiss, Germany) through an objective of 100×, NA = 1.25. The microscope is equipped with a motorized stage (Prior Scientific, UK) and with a real time image capture system integrated to a computer.

### Samples

For all applications, RBC units were obtained from Foundation of Hematology and Hemotherapy of Pernambuco (Fundação Hemope – the Ethical Committee of this Institution approved this study). All RBC units were collected in CPD-SAGM bags (Fresenius Kabi®) and stored at 4°C (±2°C). Pre storage leukocyte reduction was performed using bags with in line filters (Composelect - Fresenius Kabi®). For the zeta potential and elasticity analysis, all RBCs samples were diluted in AB serum (1∶1000 µL). Measurements of ζ were performed on the first day of each week during 36 days and at least 40 cells originated from 4 different donors were analyzed during each week. For elasticity measurements, at least 20 cells were analyzed during each week. These analyses started on day 8 and were performed until the 36th day of storage. For RBC leukodepleted samples, ζ was measured on the first day of each week during 15 days and at least 20 cells from RBC leukodepleted units were analyzed. The production of reactive oxygen species (ROS) was also quantified for standard RBCs units during the same storage period (from day 1 to day 36). For this, 10^6^ cells/mL were washed and resuspended in PBS (Phosphate Buffered Saline), incubated for 30 min with 0.5 µL DCHF-DA (dichlorodihydrofluorescein diacetate – Invitrogen) and analyzed by flow cytometry (BD FACSCalibur System – 20,000 events for each test).

### Data Analysis

To examine statistical differences or similarities presented between the groups, we use the Wilcox on rank sums test. Groups were considered significative different for p values lower than 0.05 (for a two-tail hypothesis).

### Zeta Potential Measurements

**Figure 1:**
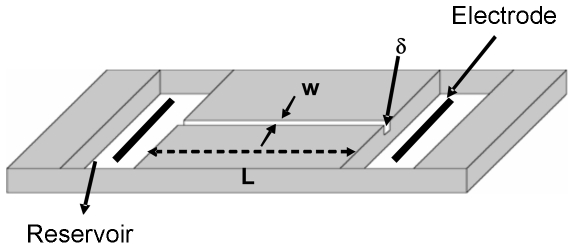
Illustration of the chamber used for zeta potential measurements.

**Figure 2:**
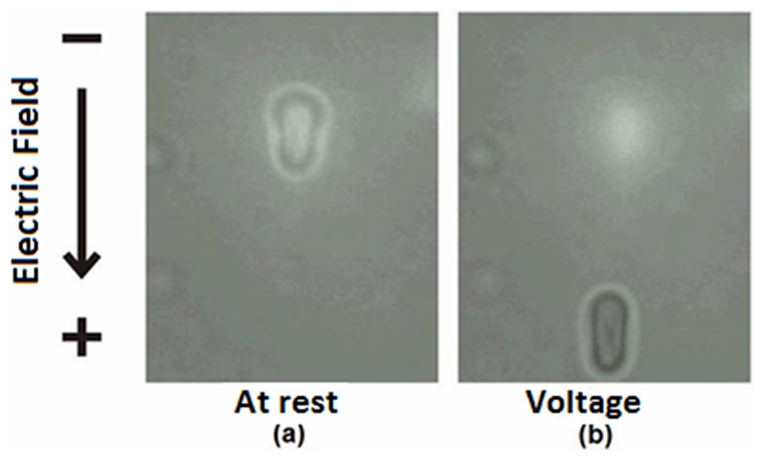
Migration of RBCs. (a) without any voltage (at rest); (b) moving in terminal velocity according to the applied voltage.

We built a special chamber for the measurements of zeta potential, consisting of two platinum electrodes (99.95%, Heraeus, São Paulo) separated by a channel of 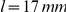 (length), 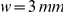 (width) and 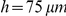 (depth) (Fig. 1). After adding RBCs to the chamber, an individual RBC was trapped with the optical tweezers while an external electrical field was applied with a voltage power supply connected to the electrodes. Because the RBC is charged and the surrounding solution is electrolytic, the RBC will move at a constant terminal speed according to the applied voltage (V) (Fig. 2). In our method each RBC was submitted to different applied voltages (30, 40, 50, 60, 70 and 80 V) and the optical tweezers were used to recapture the cell after each voltage. Therefore, the terminal velocity (ν) was measured for each applied voltage for the same cell. A plot of the terminal velocity as a function of the electrical field E (E = V/d, where d is the distance between the electrodes) allowed us to obtain the zeta potential for each cell using the Smoluchowski equation:
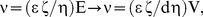
(1)where ε is the electrical permittivity of blood serum (1.06×10^−9^ C^2^/N m^2^) and η is the viscosity (1.65 cP) of the blood serum, measured with an Ostwald viscometer [24]–[26]. All data were recorded in real time and measurements of the terminal velocity were performed by video analysis with Image Pro-Plus software (Media Cybernetics, Silver Spring, MD) and Virtual Dub (by Avery Lee).

### Elasticity Measurements

To evaluate the elasticity, RBCs were added to a Neubauer chamber, captured by the optical tweezers and dragged against the blood serum with six constant velocities ranging from 140 µm/s to 290 µm/s by using the motorized stage [15]. When RBCs are dragged in blood serum they are deformed and two forces act upon the cells, a hydrodynamic force and an elastic force. Equilibrium occurs when elastic force cancels the drag force. At equilibrium,
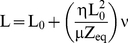
(2)where μ is the overall apparent elasticity [7], [27] and ΔL = L−L_0_ is the cell length deformation (adopting L_0_ as the cell length in the absence of any force) η is the viscosity measured using an Ostwald viscometer and ν is the velocity. The cell is located at a distance *Z_1_* from the bottom of a Neubauer chamber and *Z_2_* from the cover slip, *1/Z_eq_ = 1/Z_1_+1/Z_2_*. Therefore, the measurement of the cell length as a function of the drag velocity can be used to extract a value for μ, once the plasma viscosity η, the initial length L_0_ and *Z_eq_* are known. The cell movement at six velocities was registered by the optical tweezers camera using a video capture card in a computer. The L value was extract from video images with Image Pro-Plus software (Media Cybernetics, Silver Spring, MD). The depth *Z_1_* was measured by focusing the bottom of the Neubauer chamber and then lowering the chamber by the desired amount (in this case 50 µm) while keeping the cell fixed with the optical tweezers.

## Results

**Figure 3:**
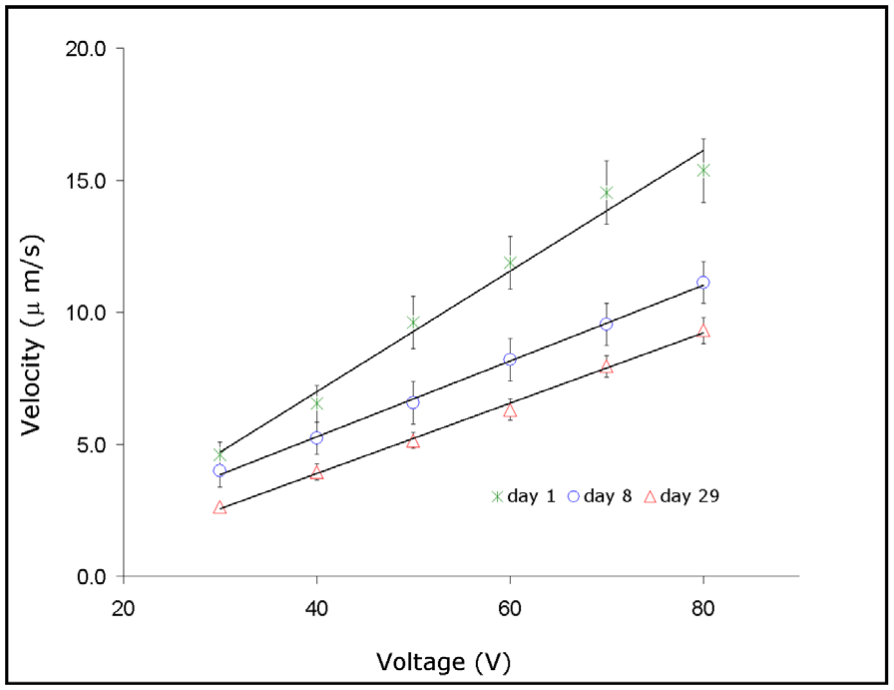
Plots of velocity against voltage as examples used to obtain the zeta potential. The higher the slope, the higher is the zeta potential. The correlation coefficients were better than 0.98. The barriers represent standard errors.

Figure 3 shows a representative plot of the velocity as a function of voltage for days 1, 8 and 29 used to obtain the zeta potential. Equation 1 shows that the slope increases with the zeta potential, therefore Figure 3 indicates that zeta potential decreases with the storage time. Table 1 shows the zeta potential measured during 36 days. On the first day of storage the ζ value was −14.5 mV, from the beginning of second week (day 8) up to the fourth week (day 22), it stabilized at an average value of −10.1 mV (p<0.001 when day 1 was compared to days 8, 15 and 22/for day 8: −9.7 mV, for day 15: −10.3 mV and for day 22: −10.2 mV), decreased to −8.5 mV during the fifth week (day 29) and remained practically constant after that (p<0.001 when day 1 was compared to days 29 and 36; p = 0.002 when the zeta potential of days 8, 15 and 22 was compared to days 29 and 36). The average zeta potential for RBCs in CPD-SAGM, therefore, decreased around 42% by the end of the storage period, compared to the cells on the first day of storage.

**Table 1:** Temporal evaluation of the zeta potential (for non leukodepleted RBCs samples) during the storage period.

Storage Time (Days)	Average Zeta Potential (mV)
Day 1	−(14.5±0.7)
Day 8*	−(9.7±0.3)
Day 15*	−(10.3±0.3)
Day 22*	−(10.2±0.4)
Day 29 to Day 36	−(8.5±0.4)

*The average zeta potential from day 8 to day 22≅−(10.1±0.3) mV.

We also measured the RBCs zeta potential after leukoreduction to evaluate the influence of enzymes released from leucocytes lysed during storage. Table 2 shows that leukodepleted RBC samples presented an analogous zeta potential decay profile to the non leukodepleted ones (the small difference for day 1 is not significative; p = 0.4). This indicates that leukodepletion does not change RBCs membrane negative charges. Furthermore, these results also show that the presence of leukocytes enzymes is not responsible for the ζ decrease (p = 0.7 when days 8 and 15 for RBCs leukodepleted samples were compared to days 8 and 15 for RBCs non leukodepleted sample).

**Table 2:** Temporal evaluation of the zeta potential (for leukodepleted RBCs samples) during the storage period.

Storage Time (Days)	Average Zeta Potential (mV)
Day 1	−(14.0±0.5)
Day 8	−(9.5±0.6)
Day 15	−(10.0±0.4)

The decay of the ζ potential was anticorrelated with the production of ROS (in standard RBCs units). DCHF fluoresces only after oxidation and is proportional to the quantity of ROS produced. While the more pronounced ζ decay was during the first week of storage (30%), the percentage of cells presenting fluorescence raised by 60% in the first week indicating an increase of around 60% in the ROS production. The percentage of fluorescent cells increased only about 16% from day 8 until day 36. Thus, flow cytometry analysis suggests that the ζ decay is caused by membrane oxidative damages.

**Figure 4:**
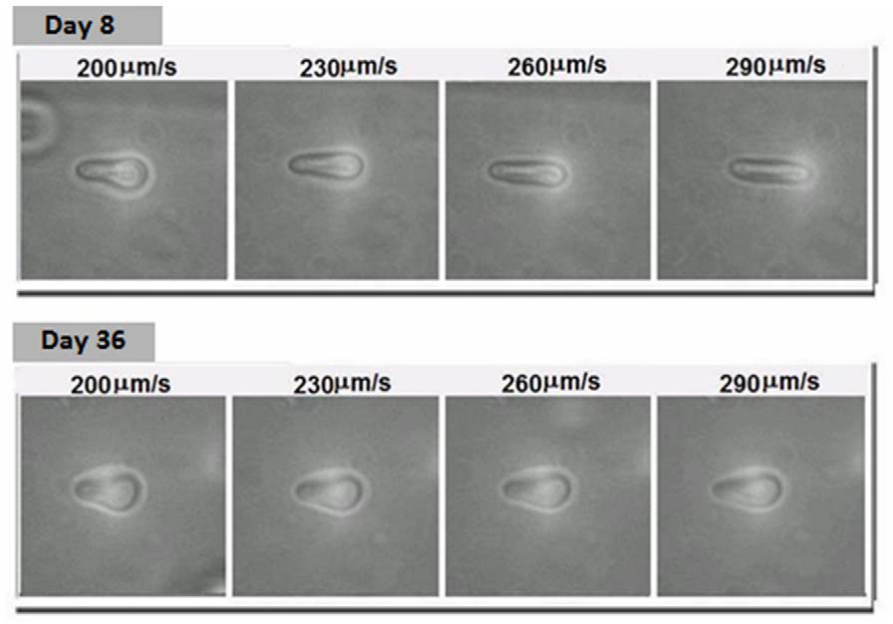
Difference between RBCs elongations according to the applied velocity in the day 8 and day 36 of storage.

**Figure 5:**
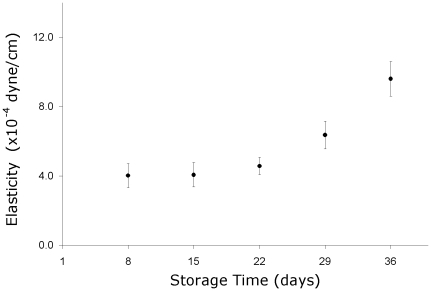
Temporal evaluation of the apparent RBC elasticity during the storage period. The barriers represent standard errors.

The loss of deformability can be easily observed by microscopic screenshots of trapped cells moving under crescent speeds in Figure 4 (this shows the difference between the elongation of cells of day 8 and of day 36 of storage). Figure 5 presents the result for RBC elasticity in CPD-SAGM as a function of the storage time. At the beginning of the second week of storage (day 8), the average elasticity value was (4.1×10^−4^±0.6×10^−4^) dyne/cm. This value presented no significant difference when compared to the elasticity (4.6×10^−4^±0.5×10^−4^) dyne/cm of day 22 (p = 0.5). On the fifth week, elasticity was (6.4×10^−4^±1.0×10^−4^) dyne/cm (p = 0.04 when compared to day 8), reaching the value of (9.6×10^−4^±1.0×10^−4^) dyne/cm at sixth week of storage (p<0.001 when compared to day 8 and p = 0.02 when compared to day 29). The apparent RBC elasticity has the same behavior of an elastic constant of a spring: the higher this value, the less elastic the RBC. Our results show that RBCs were 134% less deformable at the end of the storage period.

## Discussion

The ζ decay for non leukodepleted RBCs samples was more pronounced during the first week of storage (30%). The process of leukoreduction did not interfere in the electrical properties of the RBCs membrane and the ζ (of leukodepleted RBCs samples) presented similar decay. In other words, the results show that the ζ decay (and consequently the RBCs membrane charges decay) is not caused by the presence of leukocytes. These decays are consequence of general storage conditions related to, for example, the constituents of the preservative solutions, no matter if the RBCs came from leukodepleted samples. Furthermore, the analogous behavior observed in zeta potential and in ROS flow cytometry measurements indicates that the ζ decay is caused by oxidative damages generated by the production reactive species of oxygen.

Some reports suggested that young RBCs are more negatively charged than mature RBCs and that this could determine the mature cell sequestration in the reticulum-endothelial system [28]–[30]. Other authors reported that the removal of membrane sialic acid by sialidase enzymes (such as neuraminidase) in animal models decreased erythrocyte survival *in vivo* and the cells became more susceptible to rapid elimination from the circulation (cells were sequestered in the liver and spleen, probably due to greater adhesion among them – [31]). Moreover, endothelial cells are also negatively charged and electrostatic repulsion between RBCs and capillary walls can favor the blood flow through the microvasculature, suggesting that a loss of membrane charges can increase the adhesion of RBCs also to the capillary walls [32], [33]. These findings reported from other authors support our idea that not only elasticity, but also RBC zeta potential is an important and sensitive property that undergo changes during the storage providing new insights for transfusion purposes.

In this study we observed that there is a gradual loss of elasticity during CPD-SAGM storage. We showed that elasticity remains practically preserved until day 22, suggesting that transfusion could be more effective (mainly in critical and special cases) when RBCs stored until this fourth week are used. The most significant loss of elasticity was observed during the fifth and sixth week of storage.

Band 3 protein is associated to the elastic behavior of RBCs. The sialylated protein most plentiful in RBCs membranes is the glycophorin A, consequently this protein is one of the main responsible for the RBCs negative membrane charges. Godin and co-authors reported that RBCs presented a loss of elasticity after treatment with neuraminidase and suggested that it was caused by a closer physical connection which exists between the glycophorin A and the Band 3 proteins [4]. Therefore, the Godin and co-authors study show that changes in glycophorin A can reflect in changes in elasticity. Based on these evidences, the hypothesis of this paper is: the zeta potential decays induce a loss of cell elasticity that is observed more critically after the fourth week of storage as a consequence of oxidative damages during storage. Other supporting evidence for our hypothesis was the loss of RBCs deformability after gamma radiation observed by Brandão and co-authors [13] because it is well known that ionizing radiation can cause oxidative damages. These findings indicate that the preservation of the glycophorins integrity can be important to maintain RBCs elasticity.

In conclusion, in this study we used optical tweezers to quantify the elasticity and the zeta potential as function of storage time. We also pointed out how sensitive these two RBCs properties are to investigate RBC membrane injuries for clinical and research purposes.
